# Operon™ Platform-Enabled for Cardiometabolic Biomarker Screening and Precision Treatment Strategies: A Type 2 Diabetes-Centered Review with Cardiovascular Extension

**DOI:** 10.3390/ijms27093969

**Published:** 2026-04-29

**Authors:** Ian Jenkins, Krista Casazza, Vaishnavi Narayan, Waldemar Lernhardt, Valentina Savich, Jayson Uffens, Pedro Gutierrez-Castrellon, Jonathan R. T. Lakey

**Affiliations:** 1 GATC Health Corp., Irvine, CA 92614, USA; ian@gatchealth.com (I.J.); vaish@gatchealth.com (V.N.); waldemar@gatchealth.com (W.L.); jayson@gatchealth.com (J.U.); 2 Departments of Surgery and Biomedical Engineering, University of California Irvine, Irvine, CA 92868, USA; krista.casazza@gmail.com; 3 Elemental Translational Research SAPI, Mexico City 14357, Mexico; pedro.gutierrez@elemental.org.mx

**Keywords:** type 2 diabetes, cardiometabolic disease, biomarkers, multiomics, systems biology, artificial intelligence, machine learning, extracellular vesicles, exosomes, precision medicine, polypharmacology, cardiovascular disease

## Abstract

Cardiometabolic diseases, encompassing obesity, insulin resistance, type 2 diabetes (T2D), metabolic dysfunction-associated steatotic liver disease (MASLD), hypertension, and atherosclerotic cardiovascular disease (ASCVD), represent a vast continuum driven by multi-organ network dysregulation. Clinical risk assessment remains dominated by late-stage measures (e.g., fasting glucose, HbA1c, standard lipids). While these assessments predominate the literature and clinical trial endpoints, each incompletely capture early mechanistic risk, inter-individual heterogeneity, and differential response to interventions. Multiomics (genomics, epigenomics, transcriptomics, proteomics, metabolomics, lipidomics, microbiomics, and extracellular vesicle/exosome cargo profiling) expands the biomarker landscape but introduces translational barriers: high dimensionality, cohort heterogeneity, limited causal inference, and insufficient validation pipelines. AI-driven systems biology platforms can support cardiometabolic biomarker discovery and therapeutic translation by enabling systems-level biological inference across heterogeneous datasets, prioritizing mechanism and traceability over purely correlation-based models. GATC Health’s Operon™ platform is described as a proprietary, AI-driven internal scientific computing platform designed to support therapeutic discovery and development decision-making across the pharmaceutical lifecycle, including evaluation of drug efficacy, safety, off-target effects, pharmacokinetics (PK), pharmacodynamics (PD), and overall development risk. Operon evolved from earlier generations of GATC Health’s internal multiomic modeling systems (formerly referred to as the Multiomics Advanced Technology, MAT) and incorporates expanded data types, orchestration layers, validation workflows, and productization frameworks. Operon is operated by GATC scientists and generates structured, productized outputs (e.g., formal assessments, analyses, and decision frameworks) that are reviewed by experts. Operon methodologies have undergone internal validation and independent academic evaluation under blinded conditions, with reported classification performance (true positive rate 86% and true negative rate 91%) in controlled evaluation settings; these performance metrics should not be interpreted as guarantees of clinical success. This review provides a T2D-centered cardiometabolic biomarker landscape with cardiovascular extension and outlines how Operon-enabled multiomic integration and scenario-based simulation can support early screening, endotype stratification, mechanistic interpretation, and precision intervention design, including AI-guided polypharmacology strategies.

## 1. Introduction

Type 2 diabetes (T2D) sits at the center of the cardiometabolic continuum because it is both a sentinel phenotype of systemic network dysregulation and an accelerant of downstream organ injury through tightly coupled disturbances in glucose–lipid flux, immune–inflammatory activation, vascular biology, and kidney–heart crosstalk. Clinically, T2D confers substantially elevated risk for atherosclerotic cardiovascular disease (ASCVD), heart failure (HF), chronic kidney disease (CKD), and microvascular complications, and contemporary standards therefore emphasize routine, repeated assessment of cardiovascular and renal risk, and early use of therapies with proven cardiorenal benefit rather than a purely glucocentric approach [1]. Multiomic technologies encompass complementary measurement platforms that interrogate distinct layers of biological organization. Genomics characterizes inherited variation through genome-wide association and sequencing approaches; epigenomics captures regulatory modifications such as DNA methylation and chromatin accessibility that encode environmental exposures; transcriptomics quantifies gene expression states reflecting pathway activation; proteomics measures circulating and tissue protein abundance and signaling networks; metabolomics and lipidomics assess downstream metabolic flux and substrate utilization; and microbiome profiling evaluates host–microbe interactions and metabolite production. Each layer provides partial insight, but no single modality is sufficient to resolve the complexity of cardiometabolic disease. Increasingly, integrated multiomic approaches are being applied in cardiometabolic research to identify early disease signals, characterize biological heterogeneity, and uncover therapeutic targets, although translation into clinical practice remains limited by analytical and interpretive challenges. The mechanistic basis for this prioritization is that cardiometabolic events arise from integrated processes, adipose inflammation and impaired lipid buffering, hepatic insulin resistance with maladaptive substrate partitioning, skeletal muscle insulin resistance with impaired glucose disposal, β-cell stress/failure constraining compensation, endothelial dysfunction and prothrombotic remodeling, and neurohormonal and renal hemodynamic perturbations. Each can progress asynchronously and remain clinically “silent” while conventional glycemic and lipid measures appear acceptable. Consistent with this biology, HF has emerged as a dominant and partly glycemia-decoupled complication of T2D, supported by contemporary synthesis that links T2D to myocardial energetic remodeling, microvascular dysfunction, inflammation, sodium–fluid handling changes, and CKD-mediated amplification of HF risk [2].

**Figure 1:**
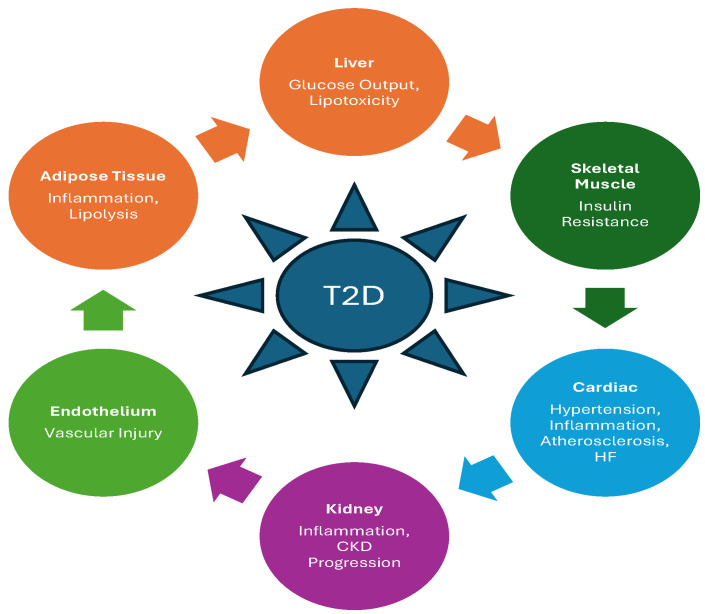
Mechanistic Network for T2D-Cenetered Cardiometabolic Disease. Type 2 diabetes (T2D) is depicted as a multi-organ network disorder involving bidirectional crosstalk among adipose tissue, liver, skeletal muscle, pancreas, vascular endothelium, and cardiorenal systems. Adipose tissue dysfunction promotes lipolysis, ectopic lipid deposition, and inflammatory cytokine release, contributing to hepatic insulin resistance, skeletal muscle glucose disposal impairment, and endothelial activation. Hepatic insulin resistance drives inappropriate glucose production and lipotoxic signaling, while skeletal muscle insulin resistance reduces postprandial glucose uptake, amplifying compensatory β-cell stress. Progressive β-cell dysfunction limits insulin secretory compensation, resulting in sustained hyperglycemia. Endothelial dysfunction and inflammatory remodeling propagate atherogenesis, thrombosis, and microvascular injury. Cardiac and renal compartments are influenced by metabolic substrate overload, hemodynamic stress, neurohormonal activation, and inflammatory signaling, contributing to heart failure (HF) and chronic kidney disease (CKD) progression. Arrows represent dynamic feedback loops rather than linear causation, emphasizing the systems-level interdependence that underlies cardiometabolic progression and residual cardiovascular risk.

Crucially, the pathobiology of T2D is heterogenous, with individuals varying markedly in the relative contributions and temporal dynamics of insulin resistance across liver, skeletal muscle, and adipose compartments; β-cell secretory capacity and stress resilience; hepatic steatosis-linked lipotoxicity; and immune–metabolic signaling, producing clinically divergent trajectories (rapid deterioration versus durable prediabetes), variable therapeutic response, and persistent residual cardiovascular risk despite guideline-concordant management (Figure 1) [3,4]. The variability in the presentation of T2D has been validated by large-scale genetic work demonstrating that common T2D risk signals cluster into biologically distinct mechanistic groupings enriched across relevant cell types (e.g., islets, adipocytes, endothelium, enteroendocrine cells), providing a framework for why a single diagnostic label (“T2D”) can mask multiple upstream causal architectures with different complication profiles [5]. In clinical practice, next-generation biomarker strategies necessitate an evolution from static, late-stage indicators to decision tools that (i) detect early mechanistic perturbations before irreversible organ damage accrues; (ii) classify clinically meaningful endotypes/subtypes; (iii) predict incident disease and complications across clinically meaningful horizons; (iv) guide individualized prevention and treatment selection (including escalation or de-escalation); and (v) monitor response and adverse biology with sensitivity to mechanism and trajectory. Multiomics, i.e., integrating genomic, epigenomic, proteomic, and metabolomic layers, can expand mechanistic and predictive resolution for early prognosis and target discovery when paired with rigorous analytics and validation, as illustrated by prospective multi-omic studies that improve incident T2D prediction and prioritize biologically plausible pathways and drug targets beyond conventional risk factors [5,6].

Multiomic translation is frequently constrained by the very features that make it powerful: high dimensionality, assay and batch variability, cohort heterogeneity, and difficulty distinguishing causal drivers from correlated downstream effects. These challenges motivate the use of AI-enabled systems biology frameworks designed to integrate heterogeneous molecular layers with clinical context and to support causal inference and actionability rather than correlation alone. Within this paradigm, GATC Health’s Operon™ (formerly Multiomic Advanced Technology [MAT™]) is an AI-driven engine that simulates human biochemistry at scale (i.e., “billions of interactions”) to enable biomarker discovery, target identification/validation, and early prediction of efficacy, toxicity, and off-target effects. Several AI-enabled platforms have been developed to integrate multiomic and clinical data for cardiometabolic and complex disease applications, broadly falling into three categories: (i) statistical multiomic integration frameworks (e.g., MOFA+, iCluster) that identify latent structure across datasets; (ii) predictive machine learning models (e.g., gradient boosting, deep neural networks) trained to optimize discrimination of clinical endpoints; and (iii) network-based systems biology approaches that model molecular interactions and pathway topology. While these approaches have demonstrated utility in biomarker discovery and risk prediction, many remain limited by either a primary focus on correlation rather than causation, limited interpretability, or insufficient translation to therapeutic decision-making. Mechanistic AI platforms, including the Operon™ system, are conceptually differentiated by explicitly modeling biological interactions and pathway dependencies to support causal inference, endotype identification, and biomarker-to-therapy translation, thereby extending beyond prediction toward structured clinical decision support.

Public-facing and internal descriptions further position Operon as an end-to-end workflow that begins with identification of diagnostic, prognostic, and monitoring biomarkers from comprehensive biological datasets and then applies mathematical modeling to evaluate causal relationships between biomarkers and pathology, thereby prioritizing validated therapeutic targets. In this review, we synthesize the cardiometabolic biomarker landscape through a T2D-centered lens and propose how Operon-enabled integration of multiomic and extracellular vesicle/exosome-derived signals can incorporate early screening, mechanistic stratification, and precision intervention design, with explicit extension to cardiovascular risk prediction and cardiometabolic outcomes.

## 2. Literature Search Strategy

This narrative review was informed by structured searches of PubMed and related biomedical databases focusing on studies published between 2015 and 2025. Search terms included combinations of “type 2 diabetes,” “cardiometabolic disease,” “multiomics,” “proteomics,” “metabolomics,” “extracellular vesicles,” “machine learning,” and “biomarkers.” Priority was given to prospective cohort studies, large-scale multiomic analyses, and mechanistic investigations relevant to biomarker discovery, disease progression, and therapeutic response. Additional references were identified through citation tracking and recent high-impact reviews. This approach was intended to capture representative advances rather than provide a systematic or exhaustive synthesis.

## 3. The Cardiometabolic Biomarker Translation Gap

### 3.1. Limitations of Current Clinical Biomarkers in T2D and Cardiovascular Risk

**Figure 2:**
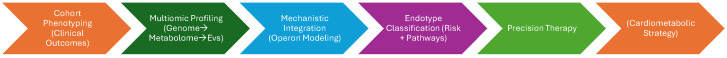
Multiomic Biomarker → Precision Pipeline. Schematic representation of a translational framework linking multiomic biomarker discovery to precision prevention and therapy selection in T2D-centered cardiometabolic disease. The workflow begins with deep cohort phenotyping (clinical characteristics, longitudinal outcomes, and comorbidities), followed by multiomic profiling (genomic, epigenomic, transcriptomic, proteomic, metabolomic, lipidomic, and extracellular vesicle (EV) layers). These heterogeneous data streams are integrated using mechanistic systems modeling to identify causal pathway drivers and biologically coherent endotypes. Endotype classification enables risk stratification across glycemic, cardiovascular, and renal domains, providing calibrated short- and intermediate-horizon forecasts. Outputs inform individualized prevention intensity (lifestyle and weight-centered strategies), early deployment of cardiorenal-protective pharmacotherapy, rational combination therapy design, and biomarker-guided monitoring. The framework emphasizes interpretability, causal inference, and prospective validation to ensure that multiomic signal translates into actionable clinical decision support rather than post hoc association.

Traditional clinical biomarkers, e.g., HbA1c, fasting plasma glucose (FPG), triglycerides, HDL-C, LDL-C, and blood pressure, are indispensable for diagnosis and routine management, yet they are often insufficient for early detection, mechanistic stratification, and individualized cardiovascular risk forecasting [7]. First, these measures largely represent late-stage integrative readouts of dysregulated physiology rather than proximal markers of causal pathway disruption. HbA1c reflects time-integrated glycemia and is influenced by factors unrelated to glucose homeostasis (e.g., erythrocyte lifespan/turnover and hemoglobinopathies), complicating interpretation across subgroups and potentially masking early metabolic deterioration in some individuals. Similarly, FPG and standard lipids incompletely reflect postprandial metabolism, hepatic substrate flux, adipose lipolysis, and tissue-specific insulin resistance, all of which may be active for years before threshold crossing into overt disease. Second, these biomarkers have limited capacity to resolve pathophysiologic heterogeneity [8]. For example, comparable HbA1c values can emerge from distinct combinations of insulin resistance and beta-cell dysfunction, and comparable LDL-C levels can occur despite divergent inflammatory and lipoprotein particle phenotypes that influence atherogenesis and residual ASCVD risk. Third, cardiometabolic outcomes (ASCVD, HF, CKD progression) are not determined solely by glycemia or standard lipids, but by coordinated inflammatory, endothelial, renal–cardiac, and neurohormonal networks; accordingly, contemporary diabetes standards emphasize systematic cardiovascular and renal risk assessment and the use of therapies with cardiorenal benefit, reflecting recognition that “glycemic control alone” does not normalize risk. Collectively, these limitations mean that patients with similar conventional panels may harbor markedly different molecular drivers, progression rates, and cardiovascular outcomes. This reflects the scenario where upstream mechanistic biomarkers and endotype-level classification would add clinical value (Figure 2).

### 3.2. Multiomics Expands Signal but Magnifies Analytical and Clinical Complexity

Multiomic profiling can, in principle, resolve the principal limitation of conventional cardiometabolic biomarkers. This is of particular salience given late-stage clinical measures collapse heterogeneous upstream biology into a small number of downstream integrators. Thus, by sampling complementary layers of disease architecture, spanning inherited susceptibility (genome), regulatory “state” shaped by exposures (epigenome), pathway activation and immune–stress programs (transcriptome), effector and signaling proteins (proteome), and real-time substrate handling and mitochondrial/redox balance (metabolome/lipidome), with additional context from host–microbe co-metabolites and immune training [9]. This breadth can expose early network shifts that plausibly precede sustained hyperglycemia and contribute to vascular complications (e.g., adipose macrophage polarization and inflammasome tone, hepatocellular lipid partitioning with ceramide/sphingolipid signaling, endothelial activation with prothrombotic remodeling, and cardiometabolic neurohormonal stress axes). Several large-scale studies illustrate the translational potential of multiomic integration in cardiometabolic disease. Integrative analyses combining genomics, proteomics, and metabolomics have identified biologically coherent pathways underlying T2D susceptibility and progression, including lipid metabolism, inflammatory signaling, and β-cell function, while improving predictive performance beyond traditional risk factors. In parallel, prospective cohort studies using metabolomic and proteomic profiling have demonstrated enhanced prediction of incident T2D and cardiovascular complications, supporting the concept that upstream molecular signatures capture disease processes not reflected in conventional biomarkers. Importantly, these studies also highlight a recurring limitation: while predictive accuracy improves, mechanistic interpretation and actionable translation often remain incomplete without systems-level modeling. Representative multiomic studies in T2D and cardiometabolic disease, including study design, biomarkers identified, and clinical implications, are summarized in Supplementary Table S1. However, the same dimensionality that increases mechanistic resolution also amplifies translation risk unless technical, temporal, and inferential constraints are explicitly engineered into study design and analytics [10]. Batch effects and pre-analytical variation (collection tubes, processing delays, storage temperature, freeze–thaw cycles, extraction chemistry, MS acquisition drift, library-prep differences) can produce artifactual structure large enough to dominate biological signal [10]. This is a well-established failure mode across high-throughput platforms and remains a major driver of non-reproducible discoveries in multi-site omics. Harmonization is further undermined by cross-platform non-equivalence (different assay chemistries, spectral libraries, peak-calling and imputation rules, vendor pipelines) and by intrinsic within-person temporal variability, particularly for transcriptomic and metabolomic layers that shift with circadian phase, feeding–fasting cycles, acute inflammation, physical activity, and medication timing, making single timepoint “omics snapshots” vulnerable to misclassification unless repeated measures, state annotation, and time-aware modeling are used [11]. Tissue specificity is an additional structural constraint: circulating blood captures systemic spillover and endocrine signals but may incompletely represent causal processes in liver, adipose, skeletal muscle, pancreas, vascular endothelium, or myocardium; large-scale tissue resources underscore the magnitude of tissue- and cell-type–specific regulation, implying that blood-only inference requires either (i) mechanistically grounded surrogate compartments (e.g., EVs), (ii) integrative latent-factor approaches, or (iii) careful triangulation with genetics and external functional data. Even when technical and temporal issues are controlled, observational multiomics is highly susceptible to confounding and reverse causation; thus, clinically relevant biomarker claims increasingly require designs that separate association from likely causality (e.g., genetics-informed analyses, longitudinal ordering, negative controls), and external validation in diverse populations to avoid ancestry-linked performance collapse and inequitable deployment [12]. As such “systems” pipelines are developed in which multiomic integration is performed with transparent structure (e.g., interpretable latent factors, pathway-level aggregation), rigorous reporting standards for prediction models (including AI/ML) to reduce bias and inflation, and explicit translation outputs (calibration, subgroup robustness, decision-curve utility, and actionable next steps) rather than post hoc statistical associations. Within a T2D-centered cardiometabolic framing, this rigor also aligns with the central premise that ML/augmented intelligence can enable earlier prognosis prior to overt clinical manifestation, but only if multiomic signal integration is coupled to reproducible pipelines, mechanistic interpretability, and prospective validation against clinically meaningful endpoints [13,14].

### 3.3. Extracellular Vesicles/Exosomes as Scalable Biomarker Reservoirs

Extracellular vesicles provide a biologically structured, scalable biomarker compartment that complements bulk plasma profiling by preserving cell-of-origin information and stabilizing RNA, protein, and lipid cargo in accessible biofluids. In T2D-centered cardiometabolic disease, EV profiling is particularly attractive for longitudinal screening because vesicle release and cargo composition respond dynamically to cellular stress states relevant to progression and complications, while remaining amenable to repeated, minimally invasive sampling. As detailed above, the clinical translation of EV biomarkers depends critically on rigorous standardization of pre-analytics, isolation, characterization, and reporting to ensure reproducibility and cross-cohort validity.

### 3.4. GATC Health Operon as a Systems Biology Platform for Biomarker-to-Target Translation

Operon is an internal scientific computing platform rather than a general-purpose analytics tool, emphasizing systems-level biological inference, integration of diverse data modalities into coherent biological representations, scenario-based simulation to explore uncertainty and risk, and interpretability and traceability of results for expert review. Operon ingests structured and semi-structured scientific data spanning multiomics (e.g., genomic, transcriptomic, proteomic, metabolomic, epigenetic), preclinical study results, clinical trial datasets, real-world evidence, and curated biological knowledge bases, and normalizes them into internal representations that enable cross-domain analysis across otherwise siloed data types. At its computational core, Operon orchestrates multiple modeling approaches (including probabilistic inference, constraint-based modeling, reinforcement learning strategies, and neural network-based pattern recognition) and evaluates effects across biological scales from molecular interactions to whole-organism phenotypes. Scenario analysis is used to explore how biological assumptions, patient characteristics, and intervention strategies may influence outcomes, informing expert-led decisions around inclusion/exclusion criteria, endpoint selection, and dosing strategies; Operon does not generate formal clinical protocols or regulatory documentation, but its outputs can inform upstream decisions. Operon also incorporates evidence synthesis workflows that aggregate signals from modeling outputs into interpretable scoring frameworks.

Operon methodologies have been described in prior evaluations of AI-based molecular prediction systems, including blinded validation studies demonstrating classification performance (e.g., sensitivity ~86%, specificity ~91%) in controlled settings. Additional internal and collaborative evaluations are ongoing to assess robustness across disease domains, input data variability, and endpoint specificity [15].

T2D and its cardiovascular sequelae arise from networked, multiorgan dysregulation that evolves over time across adipose tissue, liver, skeletal muscle, pancreas, immune compartments, vascular endothelium, and myocardium, rather than from isolated defects within a single pathway. Adipose inflammation and lipolysis alter lipid flux to liver and muscle; hepatic insulin resistance drives fasting hyperglycemia and dyslipidemia; skeletal muscle insulin resistance impairs postprandial glucose disposal; β-cell stress and failure determine durability of compensation; immune activation and endothelial dysfunction accelerate atherogenesis, and myocardial metabolic remodeling contributes to HF risk. These processes interact nonlinearly, exhibit redundancy and compensation, and vary in relative dominance between individuals and across disease stages, which explains why single biomarkers or unidimensional risk models often fail to predict progression or cardiovascular outcomes reliably. Mechanistic AI is valuable beyond management of large datasets extending to capability, when meticulously designed, of integrating heterogeneous multiomic layers into coherent, biologically grounded pathway models that reflect this system’s architecture. Such models enable inference of causal drivers rather than mere correlates by embedding molecular data within mechanistic networks, thereby distinguishing upstream regulators from downstream consequences and transient signals from stable disease-defining processes. This approach identifies biologically meaningful endotypes, e.g., inflammation-dominant, lipotoxic-dominant, β-cell-vulnerable, or vascular-injury-predominant phenotypes that carry distinct trajectories of glycemic deterioration and cardiovascular risk despite similar clinical presentations. Mechanistic AI further derives value by explicitly linking biomarker patterns to intervention strategy selection, aligning dominant pathways with preventive or therapeutic levers (e.g., weight-centric strategies, insulin sensitization, anti-inflammatory modulation, or early cardiorenal-protective therapies), rather than treating prediction as an endpoint. Finally, by encoding causal structure and pathway logic, mechanistic AI can inform validation design and risk stratification for both clinical trials and real-world deployment. Collectively, mechanistic AI promotes enriched cohort selection, subtype-specific endpoint choice, calibration across populations, and interpretation of treatment effects; thereby bridging the persistent gap between molecular discovery, clinical prediction, and actionable precision care in T2D-centered cardiometabolic disease.

## 4. Biomarkers for Early Dysglycemia, Insulin Resistance, and Preclinical Progression

Current clinical biomarkers used to define dysglycemia and cardiometabolic risk (i.e., fasting plasma glucose, 2-h oral glucose tolerance test [OGTT] glucose, HbA1c, fasting insulin–derived indices [e.g., HOMA-IR], blood pressure, and standard lipids) remain indispensable for diagnosis, staging, and guideline-based management, but they are intrinsically “late” readouts that collapse multi-organ pathophysiology into downstream integrators [1]. In the years preceding diagnostic thresholds, insulin resistance can be substantial yet clinically occult because β-cells initially compensate via hyperinsulinemia, maintaining near-normal fasting glucose and modest HbA1c while postprandial dysmetabolism, impaired peripheral glucose disposal, and maladaptive substrate partitioning advance (i.e., a high-risk metabolic state can exist in “normoglycemia”). Mechanistically, identical HbA1c or fasting glucose values can arise from very different causal mixtures: hepatic insulin resistance with inappropriate endogenous glucose production; skeletal muscle insulin resistance with reduced glucose disappearance; adipose tissue insulin resistance with accelerated lipolysis, ectopic lipid deposition, and adipokine/inflammatory remodeling; incretin-axis perturbations that alter meal-stimulated insulin secretion and glucagon dynamics; and progressive β-cell secretory stress with loss of disposition (the coupled relationship between insulin secretion and insulin sensitivity) [1]. The absence of know etiology matters because cardiovascular and renal outcomes are driven not only by glycemic exposure and LDL-C but also by vascular inflammation, endothelial dysfunction, lipotoxic and ceramide signaling, oxidative and ER stress, autonomic/neurohormonal activation, and kidney–heart cross-talk, pathways that can remain active despite “controlled” conventional panels and help explain residual ASCVD and heart-failure risk in treated T2D [16]. Contemporary standards therefore emphasize earlier, multifactorial cardiometabolic risk reduction and the use of therapies with proven cardiovascular and kidney benefit in high-risk patients, reinforcing the need for biomarkers that detect upstream biology rather than simply confirm downstream dysglycemia.

Integrating mechanisms into biomarker strategy reframes early dysglycemia, detectable before sustained hyperglycemia, where multiomic signatures can (i) identify compensated insulin resistance prior to diagnostic cut points, (ii) partition insulin resistance into hepatic-, muscle-, and adipose-predominant endotypes using pathway-driven signals (lipid flux/steatosis biology, mitochondrial overload, immune–metabolic activation, and endocrine stress programs), and (iii) forecast near-term “conversion risk” from prediabetes to T2D and downstream cardiometabolic events with clinically actionable time horizons [17]. Human physiology studies have demonstrated that distinct defects in fasting versus postprandial control map onto different underlying mechanisms (e.g., impaired glucose disappearance and β-cell dysfunction as key contributors to postprandial hyperglycemia in early dysglycemia), highlighting why static fasting measures alone can miss high-risk states. At population scale, prospective data also show that targeted NMR metabolomic profiling adds prognostic information for incident T2D beyond conventional risk factors, consistent with the concept that upstream metabolic networks (lipoprotein subfractions, amino acid and inflammatory glycoprotein signals, ketone-related markers, and fatty-acid patterns) can improve discrimination and calibration when integrated into risk models [18]. Complementary evidence from real-world electronic medical record cohorts indicates that machine-learning models can improve prediction of progression from prediabetes to diabetes, underscoring that clinically relevant forecasting is feasible when longitudinal phenotypes and covariate depth are leveraged appropriately. In high-rigor programs, these opportunities must be matched by design discipline: prospective time-to-event modeling with prespecified endpoints; explicit handling of fasting status, diurnal and postprandial variability, and weight-change/medication confounding; subgroup calibration across age, sex, ancestry, and kidney-function strata; and transparent reporting of incremental utility over established predictors (discrimination, calibration, reclassification, and decision-curve analyses), so that added molecular resolution translates into reproducible clinical action rather than irreproducible association.

Genomic biomarkers provide a stable view of inherited liability that shapes the cardiometabolic life course, spanning β-cell development and secretory capacity, insulin signaling and glucose transport, adipocyte differentiation and lipid storage “expandability,” hepatic lipid handling, innate/adaptive immune set-points, and vascular remodeling pathways [19]. Because germline variation is time-invariant, it is uniquely suited for lifetime risk estimation, early prevention intensity decisions, and prospective stratification long before conventional biomarkers cross diagnostic thresholds [20]. However, clinical yield of genomic biomarkers is fundamentally conditional on (i) the ancestry composition of the discovery GWAS [21] and (ii) the extent to which inherited predisposition is expressed as active, measurable pathway dysregulation in a given environment (obesity, diet quality, physical activity, sleep/circadian disruption, and medication exposures) [22]. Large multi-ancestry evaluations demonstrate that T2D polygenic risk score performance varies by clinical context (e.g., age, obesity, hypertension status), underscoring that PRS should be interpreted as genetic liability interacting with current physiology, not deterministic [23]. Practically, genomics becomes most actionable when integrated with dynamic layers (i.e., metabolomics, lipidomics, proteomics, EV cargo, and longitudinal clinical trajectories) that quantify whether genetic risk has translated into dominant, currently active mechanisms (e.g., hepatic insulin resistance with dysregulated lipid flux, adipose inflammation with immune activation, or β-cell stress with impaired proinsulin processing) [24]. In the cardiovascular extension, genomics can add incremental resolution for residual risk: a genome-wide coronary heart disease (CHD) polygenic risk score improved prediction of major adverse cardiovascular events among people with T2D beyond a clinical risk score [25], supporting the concept that inherited vascular susceptibility can refine who most benefits from earlier or more intensive cardiorenal-protective strategies [25]. At the same time, the field’s most rigorous translational use of genomics is not limited to prediction; it also strengthens causal inference and target prioritization through Mendelian randomization and colocalization approaches that help discriminate causal drivers from correlated biomarkers in observational cardiometabolic cohorts, particularly relevant where adiposity, glycemia, lipids, inflammation, and kidney function are tightly intercorrelated [26]. Finally, clinical implementation demands explicit acknowledgment of current limitations: recent expert review concludes that, to date, T2D polygenic scores are often outperformed by routine clinical measures for near-term prediction, and broad utility will require improved multi-ancestry representation, harmonized evaluation, and, importantly, integration with dynamic biomarkers that capture mechanistic state and modifiable risk [27].

## 5. Pharmacogenomics and Treatment Response Variability

Pharmacogenomic variation contributes to interindividual differences in drug metabolism, efficacy, and safety, representing an important dimension of precision medicine in T2D and cardiometabolic disease. Variants in genes involved in drug transport, metabolism (e.g., cytochrome P450 enzymes), and molecular targets can influence response to commonly used therapies, including metformin, sulfonylureas, and newer cardiometabolic agents. However, pharmacogenomic signals alone rarely explain the full variability in treatment response, which is shaped by broader metabolic, inflammatory, and organ-specific contexts. Accordingly, integration of pharmacogenomics with multiomic and clinical data may provide a more complete framework for individualized therapy selection.

Epigenomic regulation, encompassing DNA methylation, chromatin accessibility, histone post-translational modifications, and higher-order chromatin architecture, provides a biologically plausible “molecular memory” layer that can link cumulative exposures to durable changes in gene regulatory programs relevant to insulin resistance, β-cell vulnerability, vascular inflammation, and end-organ remodeling. In cardiometabolic disease, repeated or transient metabolic stressors (hyperglycemia, dyslipidemia, oxidative stress, cytokine signaling, hypoxia, and altered nutrient sensing) can be translated into persistent transcriptional bias through stable epigenetic marks at promoters/enhancers governing inflammatory signaling (e.g., NF-κB–linked programs), fibrotic remodeling (TGF-β–associated pathways), mitochondrial and redox regulation, endothelial nitric oxide biology, and maladaptive cellular stress responses. The clinically observed phenomenon that complication risk may remain elevated despite later improvement in glycemia, i.e., “metabolic memory” and positions epigenomic features as both mechanistic mediators and biomarkers of long-horizon cardiometabolic risk, particularly for microvascular and renal outcomes where persistent inflammatory–fibrotic programming is pathophysiologically plausible [28].

Epigenomics is especially attractive because it can (i) encode exposure history (adiposity, diet quality, smoking, inflammation, environmental stressors), (ii) plausibly participate in durable pathway “set points” that shape future metabolic responses, and (iii) offer a bridge between early-life programming and adult cardiometabolic trajectories under the developmental origins of health and disease paradigm. However, the same properties that make epigenomics compelling also impose stringent rigor requirements. First, tissue specificity is non-negotiable: whole-blood methylation is not a direct proxy for epigenetic states in hepatocytes, adipocytes, myocytes, pancreatic islets, vascular endothelium, or renal parenchyma, and cell-mixture effects in blood can masquerade as disease signals if not explicitly modeled. Second, assay harmonization and batch effects are major threats to reproducibility given platform differences (array vs. sequencing), pre-analytic variability, and normalization choices. Third, causal interpretation is challenging because epigenetic marks can be both causes and consequences of metabolic derangement, and reverse causality is common when profiling occurs after disease onset. The most defensible designs therefore prioritize prospective and longitudinal sampling anchored to hard outcomes, and they explicitly evaluate incremental value beyond established predictors using time-to-event models, calibration, and external replication. This rigor standard is feasible: blood-based EWAS meta-analysis across multiple prospective cohorts has identified methylation sites measurable 7–10 years before diagnosis associated with incident T2D [29], and independent prospective work in EPIC-Norfolk similarly supports incident T2D–associated methylation variation years before onset [30]. Beyond single-site associations, recent approaches that aggregate CpGs into methylation-based risk scores have shown improvement in 10-year incident T2D prediction beyond standard risk factors, illustrating the clinical direction of travel toward deployable epigenomic risk stratification rather than purely descriptive biology [31]. For cardiometabolic extension, contemporary synthesis in Circulation Research emphasizes that epigenetic mechanisms are already implicated in early cardiovascular and metabolic disease development and may support precision-medicine strategies, while underscoring that translation demands better mechanistic perturbation tools, multi-omic integration, and equitable cohort representation [32].

Transcriptomic signatures can capture active pathway states that often precede overt dysglycemia: immune activation (innate/adaptive), ER stress, oxidative stress, mitochondrial response programs, adipose remodeling, and endothelial inflammatory signaling. These states are relevant not only to T2D onset but also to ASCVD risk via endothelial dysfunction and immune-mediated atherogenesis. The main translational constraints are temporal variability (transcriptomes change with infection, stress, circadian rhythms), tissue specificity (blood vs. target tissue), and pre-analytical variability. Two mitigation strategies consistent with high scientific rigor are: (i) repeated sampling (trajectory-based modeling rather than single timepoints) and (ii) use of structured compartments as a stable, physiological source of RNA cargo reflecting cellular stress and tissue communication.

Proteomics provides a high-throughput view of circulating endocrine regulators (adipokines, hepatokines), inflammatory mediators, immune signaling, coagulation/complement systems, and tissue injury markers [33]. In a T2D-centered framework, proteomic profiles can (i) detect inflammatory and endothelial injury endotypes associated with accelerated progression; (ii) refine microvascular and macrovascular risk (peripheral artery disease [PAD], coronary heart disease [CHD] and HF) beyond standard panels; and (iii) serve as monitoring biomarkers for response or adverse biology (e.g., pro-inflammatory states despite glycemic improvement). Emerging large-cohort studies underscore clinical plausibility: recent UK Biobank proteomic analyses have reported shared proteomic signatures between T2D and coronary heart disease and identified subsets of proteins with evidence consistent with causal associations, illustrating how plasma proteomics may bridge metabolic disease biology to vascular outcomes. In parallel, Diabetes Care recently reported that large-scale plasma proteomics improves prediction of incident PAD in individuals with T2D, highlighting a practical example of proteomics refining vascular complication risk stratification in a real-world cohort setting.

Metabolomics captures integrated physiology: substrate handling, hepatic glucose output, mitochondrial overload, branched chain/aromatic amino acid metabolism, lipid oxidation, and intermediary pathway disruption. Because metabolite levels can respond early to insulin resistance and hepatic lipid flux changes, metabolomics is well positioned for preclinical detection and monitoring of intervention response (diet, exercise, weight loss therapies, insulin sensitizers, GLP-1RA/SGLT2i strategies), provided that pre-analytical variability is tightly controlled. Large prospective evidence supports value. For example, in UK Biobank, circulating metabolomic biomarkers detectable by nuclear magnetic resonance (NMR) improved prediction of T2D beyond conventional risk factors, demonstrating feasibility for population-scale deployment [17]. To date, metabolic biomarkers are limited by fasting and postprandial standardization (or explicit modeling of state), dietary and medication covariates, harmonization across platforms, and biologically interpretable mapping (e.g., pathway enrichment tied to hepatic IR vs. muscle IR vs. inflammatory stress).

Lipidomics resolves lipid species (beyond triglycerides/LDL-C) that reflect hepatic lipid flux, adipose spillover, membrane remodeling, sphingolipid/ceramide pathways, and inflammatory lipid mediators implicated in insulin resistance and beta-cell stress. In T2D, lipidomic signatures can identify lipotoxic endotypes where hepatic steatosis and ectopic lipid deposition contribute to insulin resistance and progressive beta-cell dysfunction. For CVD extension, lipidomics can quantify inflammatory lipid patterns plausibly linked to atherogenesis and residual ASCVD risk beyond LDL-C, supporting mechanistic refinement of “residual risk” populations. Translational challenges include assay complexity, cross-lab reproducibility, and the need to distinguish causal lipid mediators from correlated species; robust designs incorporate targeted lipid panels with validated analytic pipelines and external replication.

Across these biomarker classes, the scientific bar for clinical translation is now well-defined: models must be developed in prospective, time-to-event frameworks with prespecified endpoints and horizons; pre-analytical state effects (fasting/postprandial timing, diet, circadian effects, acute illness) and key confounders (medications, weight change, kidney function, systemic inflammation) must be controlled or explicitly modeled; and performance must be reported not only as discrimination (Area Under the Curve/C-statistic) but also as calibration, subgroup robustness across ancestry/sex/age strata, incremental value over established predictors (reclassification and decision-curve analyses), and perhaps most importantly, applicability (clear mapping from biomarker-defined endotype to prevention/treatment selection and monitoring cadence). Within this rigor framework, ML/AI-enabled multiomic integration is most valuable when it moves beyond correlation to mechanism-informed stratification that can support individualized prevention intensity and cardiometabolic outcome reduction.

### Microbiome-Related Biomarkers and Host–Microbe Metabolic Crosstalk

Microbiome-related biomarkers are increasingly relevant to T2D-centered cardiometabolic stratification because the gut ecosystem sits upstream of multiple potential metabolic control points, intestinal barrier integrity and endotoxemia, innate immune priming, enteroendocrine signaling, and bile acid–FXR/TGR5 pathways that regulate hepatic gluconeogenesis, lipid handling, and insulin sensitivity, while also generating circulating metabolites that can access the liver, vasculature, adipose tissue, and skeletal muscle through the portal and systemic circulations. Dysbiosis-associated shifts in short-chain fatty acid (SCFA) production, altered bile acid pools and microbial bile acid transformations, and enrichment of pro-inflammatory microbial products (e.g., LPS and other pathogen-associated molecular patterns) can converge on insulin receptor signaling, adipose macrophage activation, hepatic glucose output, and endothelial inflammatory tone, providing a mechanistic basis for why paired “composition + function” readouts (taxonomy plus metabolite panels) may outperform either layer alone as biomarkers of preclinical dysglycemia, insulin resistance endotypes, and downstream vascular risk. In a clinically actionable framing, microbiome-derived signatures become most useful when they are explicitly linked to modifiable levers—fiber quality and fermentability, dietary fat composition, weight loss trajectories, and medication exposures (notably metformin)—and when they are anchored to measurable host pathways (e.g., bile acid signaling modules, inflammatory markers, and metabolomic flux signatures) rather than treated as isolated ecological associations [34,35,36].

The principal barrier to clinical-grade deployment is the microbiome’s high context sensitivity: geography, habitual diet, circadian patterning, intercurrent infection, antibiotics, GLP-1RA/SGLT2i and other medication use, bowel transit time, and sampling/processing variation can each dominate variance and create spurious “biomarkers” that fail replication. Accordingly, rigorous microbiome biomarker programs should be designed like other high-dimensional clinical assays: standardized protocols (collection medium, time-to-freeze, storage conditions, DNA extraction and library chemistry), pre-specified covariates (dietary pattern, recent antibiotics, metformin and other agents, renal function, BMI/weight change), and repeated measures that model within-person stability and trajectory rather than single timepoints. Importantly, clinical utility should be judged by incremental value over established risk engines (calibration, decision-curve analysis, and net reclassification), and by whether microbiome-informed stratification predicts response to diet or lifestyle intervention rather than merely correlating with baseline phenotype; the feasibility of integrating microbiome features into predictive models for individualized glycemic responses has been demonstrated in large human cohorts using machine-learning approaches coupled to dense phenotyping [37].

## 6. Extracellular Vesicles (EVs)

EVs, including small EVs often operationally termed “exosomes”, are increasingly viewed as biomarker reservoirs for T2D-centered cardiometabolic screening because they sit at the interface of cellular stress biology and inter-organ communication. In insulin resistance and evolving dysglycemia, multiple tissues (adipose, liver, skeletal muscle, pancreas, endothelium, myocardium, kidney) experience convergent stressors, e.g., nutrient excess, lipotoxicity, oxidative stress, endoplasmic reticulum (ER) stress, mitochondrial overload, and innate immune activation, that remodel vesicle biogenesis, cargo sorting, and vesicle release. EV cargo (miRNAs and other RNAs, proteins, lipids, metabolites) is not merely “spilled” from plasma; rather, it can reflect regulated packaging pathways (e.g., ESCRT-dependent/independent mechanisms, tetraspanin-enriched microdomains, lipid raft dynamics) that are sensitive to cytokine signaling and metabolic state. Compared with bulk plasma analytes that average signals across tissues, EV profiling can function as a biologically structured compartment that partially mitigates tissue-specificity constraints, enabling repeated, minimally invasive sampling to track pathway drift over time and to capture early transitions from compensation (hyperinsulinemia with normoglycemia) to decompensation (β-cell failure with sustained hyperglycemia). Broad syntheses in diabetes emphasize EVs as multiorgan crosstalk mediators linking insulin resistance, inflammation, endothelial injury, and downstream complications, supporting their inclusion in T2D biomarker architectures that explicitly include ASCVD/HF/CKD endpoints [38,39,40].

Mechanistically, EVs can propagate or amplify cardiometabolic injury by transferring functional cargo that reprograms recipient cells. A compelling human-relevant example for CVD extension is the demonstration that erythrocyte-derived EVs in T2D carry arginase-1 and induce endothelial dysfunction via reduced NO bioavailability and increased oxidative stress, with attenuation of vascular dysfunction upon arginase or oxidative stress inhibition, directly connecting a circulating EV compartment to a canonical vascular injury pathway in T2D [41]. In parallel, disease-area reviews of diabetic cardiomyopathy describe EVs as plausible mediators and indicators of myocardial metabolic remodeling, fibrosis, inflammation, and impaired angiogenic signaling, features that align with the HF phenotype that increasingly dominates cardiovascular morbidity in long-standing T2D [42]. These mechanistic linkages strengthen the rationale for EVs in screening: EV assays can be designed not only to correlate with outcomes, but to quantify pathway-concordant cargo modules (e.g., endothelial NO/ROS axis; inflammatory cytokine programs; fibrotic signaling; lipid-handling signatures) that map to actionable endotypes and cardiometabolic complication risk.

The principal barrier to EV biomarker translation is methodological sensitivity, which can easily generate irreproducible signals if preanalytics and isolation/characterization are not tightly controlled. The ISEV MISEV2018 guidance sets minimum information and reporting standards (sample handling, EV separation approach, EV identity characterization, and appropriate controls, including assessment of non-EV fractions), effectively making “rigor-by-design” a prerequisite for credible EV claims in cardiometabolic cohorts [43]. In practice, EV deployment for T2D-centered cardiometabolic screening would therefore benefit by specification of: standardized collection conditions (fasting state, time-of-day, anticoagulant choice, processing delays, freeze–thaw limits), harmonized isolation (e.g., SEC/ultracentrifugation/immunocapture choices aligned to intended EV subtypes), orthogonal characterization (particle counts, protein markers, morphology), and cross-platform replication. Reviews focused on diabetic vascular complications underscore that endothelial EV biology is tightly coupled to vascular dysfunction and that EVs are plausible diagnostic and therapeutic targets, but they also reinforce that assay standardization and mechanistic anchoring are central to clinical scalability [44].

## 7. From Biomarkers to Treatment Strategies: Precision, Prevention and Therapy

A clinically useful biomarker strategy in T2D-centered cardiometabolic disease must extend well beyond risk prediction to enable decision-making that alters clinical trajectory (Table 1). At minimum, biomarkers must discriminate individuals who are likely to progress from compensated dysmetabolism to overt T2D and those at heightened risk for downstream complications such as ASCVD, heart failure, and CKD, because these outcomes, not glycemia alone, drive morbidity and mortality. Regulatory frameworks explicitly recognize this distinction: the FDA–NIH Biomarkers, EndpointS, and other Tools (BEST) Resource defines clinically useful biomarkers by their ability to characterize disease risk or presence as well as their context of use, including prognostic, predictive, monitoring, and response-guiding applications [45]. A biomarker’s value is realized when it informs what action should be taken, for whom, and with what intensity. In cardiometabolic disease, this includes guiding the timing and aggressiveness of lifestyle intervention, the selection and escalation of weight-management pharmacotherapy, and early deployment of agents with proven cardiovascular and renal benefit in individuals whose molecular and clinical profiles indicate disproportionate risk. This approach is fully aligned with contemporary diabetes care paradigms, which integrate glycemic control with weight management and cardiorenal risk reduction in structured, person-centered treatment pathways rather than linear, glucose-threshold-driven algorithms [1].

Coupling multiomic biomarkers with AI/ML frameworks capable of integrating heterogeneous molecular and clinical signals into calibrated prognostic and mechanistic models underlies translation. Such approaches can identify risk states and disease endotypes prior to clinical manifestation, when prevention is most effective and organ injury may still be reversible, thereby reframing screening from passive detection to proactive intervention design. Biomarkers become instruments of precision prevention and therapy selection: they support stratified deployment of interventions, rational sequencing or combination of therapies in a network disease, and dynamic monitoring of response and emerging risk. The practical implication is a shift from one-size-fits-all treatment escalation toward biologically informed prevention and precision therapy, where the success of a biomarker is judged not by statistical association alone, but by its ability to improve outcomes through earlier, more targeted, and more durable cardiometabolic risk reduction [46].

## 8. Precision Pharmacotherapy Selection

Precision pharmacotherapy selection in T2D is increasingly defined by event prevention (ASCVD, heart failure, CKD progression) rather than glycemic control alone, because cardiometabolic outcomes emerge from interlocking mechanisms that vary in relative dominance across individuals and across time. Contemporary standards therefore prioritize a comorbidity-first strategy in which glucose-lowering agents with proven cardiorenal benefit are deployed based on ASCVD/HF/CKD phenotype and risk, not solely HbA1c or background metformin use, reflecting the reality that “controlled” glycemia can coexist with persistent vascular inflammation and myocardial/renal vulnerability [1]. The ADA/EASD consensus approach, explicitly frames therapy selection around person-centered factors (weight, hypoglycemia risk, comorbidities, complications, and individualized treatment goals) and supports early use of SGLT2 inhibitors and GLP-1 receptor agonists when cardiorenal risk is present [47,48]. Mechanistically, SGLT2 inhibition produces benefits that extend beyond glucose lowering via renal tubular effects (natriuresis, restoration of tubuloglomerular feedback, reduced intraglomerular pressure), systemic hemodynamic unloading, and improved myocardial energetics and congestion biology. These effects are consistent with broad benefits in CKD and heart failure phenotypes. The EMPA-KIDNEY trial demonstrated reduced risk of kidney disease progression or cardiovascular death in a broad CKD population, supporting early cardiorenal risk-directed selection even when glycemia is not the primary determinant [49]. Similarly, dapagliflozin improved outcomes in heart failure with mildly reduced or preserved ejection fraction in DELIVER, underscoring that HF-risk biology is partly decoupled from glycemic severity and can be modified through pathway-relevant pharmacology [50]. In parallel, GLP-1 receptor agonism exerts weight-, appetite-, and cardiometabolic effects with pleiotropic vascular and inflammatory modulation; the SELECT trial showed semaglutide [47,48] mg reduced major adverse cardiovascular events in individuals with overweight/obesity and established CVD without diabetes, strengthening the rationale for weight- and inflammation-linked therapeutic targeting as a cardiovascular prevention strategy adjacent to T2D care [51]. Collectively, these data highlight why a biomarker-informed, endotype-based paradigm is needed: individuals with inflammation-predominant, lipotoxic-predominant, β-cell-vulnerable, or vascular-injury-predominant biology may require different first-line combinations, different escalation thresholds, and different monitoring cadence to reduce both glycemic deterioration and residual cardiometabolic risk [46]. Emerging evidence supports the application of AI-enabled frameworks in pharmacotherapy optimization. Machine learning models incorporating clinical and molecular data have demonstrated improved prediction of drug response and adverse events across multiple therapeutic domains, including cardiometabolic disease. In parallel, network-based and systems pharmacology approaches have been used to identify synergistic drug combinations and repurpose existing agents by mapping drug effects onto disease-relevant pathways. These approaches align with mechanistic AI paradigms in which therapeutic selection is guided by pathway-level dysregulation rather than single biomarker thresholds, providing a foundation for rational combination therapy in complex, network-driven diseases.

Cardiometabolic disease is also a network disorder. Thus, single-node interventions may yield partial benefit that is attenuated by redundancy and compensatory pathway activation, while real-world polypharmacy increases the likelihood of adverse events and drug–drug interactions, particularly in multimorbidity (HF, CKD, MASLD/MASH) where renal clearance, volume status, and hemodynamics constrain options. Mechanistic AI is most impactful here when it can (i) formalize biomarker patterns into mechanistic pathway hypotheses; (ii) distinguish causal drivers from downstream correlates; and (iii) rationalize combination therapy selection and sequencing as a systems-control problem rather than an additive checklist. In practice, this requires algorithms that integrate clinical trajectories with multiomic signals to identify dominant dysregulated modules, propose intervention sets predicted to shift those modules toward a healthier microenvironment, and quantify uncertainty and safety risk before clinical testing. This represents an approach aligned with the broader objective of prognosing T2D progression prior to clinical manifestation and translating biomarker-defined endotypes into prevention and treatment intensity decisions with explicit cardiovascular extension.

## 9. Operon as the “Integrator” Across This Landscape

A pragmatic implementation pathway for T2D-centered cardiometabolic screening with cardiovascular extension begins with cohort phenotyping and sample architecture that mirrors guideline-driven risk assessment while adding scalable molecular resolution. Core clinical variables (demographics, anthropometrics, vitals, standard laboratories, medication exposure, family history, and comorbidities) should be captured alongside longitudinal endpoints spanning incident T2D, ASCVD events, heart failure, and CKD progression to support time-to-event modeling and clinically meaningful prediction horizons. This clinical layer can be paired with multiomics, one-time genome/exome profiling integrated with serial proteomics, metabolomics, and lipidomics, and complemented by EV/exosome profiling using standardized isolation, characterization, and cargo measurement (RNA/protein/lipid classes) to ensure reproducibility and interpretability. Multiomic integration and causal inference can then be performed using Operon, via normalizing heterogeneous scientific data into internal representations and orchestrating multiple computational modeling approaches across biological scales to enable integrated biological reasoning and scenario-based simulation. Deployable outputs should include short-horizon risk estimates (e.g., 12–36 months) for T2D progression and ASCVD/HF events, endotype/subtype classification, mechanistic pathway attribution, intervention and monitoring recommendations aligned to clinical context, and explicit uncertainty/data-completeness metrics. Finally, cardiometabolic deployment requires endpoint-specific validation across diverse cohorts and prospective designs. A summary of available validation studies and performance characteristics of the Operon platform is provided in Supplementary Table S2.

## 10. Limitations and Implementation Challenges

Despite its conceptual advantages, the proposed multiomic–AI framework faces several important limitations. First, clinical implementation requires substantial infrastructure for data acquisition, storage, and integration, which may limit scalability in resource-constrained settings. Second, cost-effectiveness remains uncertain, particularly for serial multiomic profiling, and will depend on demonstration of improved clinical outcomes and healthcare utilization. Third, model generalizability depends on diverse and representative training datasets; without this, performance may degrade across populations. Fourth, interpretability and regulatory acceptance of AI-driven decision frameworks remain evolving challenges, particularly in high-stakes clinical contexts. Finally, prospective validation linking biomarker-guided decision-making to improved cardiometabolic outcomes is essential before widespread adoption.

## 11. Conclusions

T2D-centered cardiometabolic disease should be treated (and studied) as a systems disorder in which metabolic, inflammatory, vascular, renal, and myocardial pathways co-evolve through nonlinear feedback across adipose tissue, liver, skeletal muscle, pancreas, immune compartments, endothelium, and myocardium. The “so what” is that late-integrating clinical biomarkers (glycemia and standard lipids) remain indispensable for diagnosis and monitoring, yet they are structurally mismatched to the clinical objectives that matter most: identifying high-risk individuals before irreversible organ injury, distinguishing mechanistically distinct endotypes that drive heterogeneous progression, and selecting therapies and monitoring strategies that reduce ASCVD, HF, and CKD outcomes rather than simply normalizing glucose. Multiomics provides the upstream resolution needed to measure the relevant biology, but its clinical translation depends on overcoming a consistent set of barriers: high dimensionality, tissue specificity, temporal variability, cohort heterogeneity, and the difficulty of separating causal drivers from downstream correlates. The implication is that next-generation cardiometabolic screening and precision therapy will not be won by adding “more biomarkers” alone; it will require integrated, mechanism-aware inference that converts multiomic patterns into interpretable pathway hypotheses, endotype classification, calibrated risk estimates, and testable intervention strategies with explicit uncertainty bounds.

An essential requirement for successful translation is the development and validation of models in diverse, globally representative populations. Many existing genomic and multiomic datasets remain disproportionately derived from populations of European ancestry, raising concerns regarding generalizability and equity. Ensuring that multiomic and AI-driven approaches are trained and validated across diverse ancestry, socioeconomic, and clinical contexts is critical to avoid exacerbating health disparities. In parallel, improving accessibility and affordability of advanced molecular profiling and AI-enabled analytics will be necessary to ensure that precision cardiometabolic medicine can be deployed broadly rather than remaining limited to specialized settings.

Operon is an internally operated, mechanistic AI platform intended to support precisely these translation steps, integrating heterogeneous data into physiologically relevant models, enabling causal mapping to prioritize drivers over correlates, and producing structured outputs that can be reviewed by scientific experts and used to inform biomarker selection, target prioritization, and trial strategy. The practicality of cardiometabolic medicine is twofold. First, an Operon-enabled workflow can make early detection actionable by tying risk and endotype calls to concrete prevention and treatment pathways (who needs intensified weight-centered therapy; who is lipotoxicity-dominant and likely to benefit from flux-reducing strategies; who is inflammation/vascular-injury dominant and needs earlier cardiovascular risk modification; who is β-cell-vulnerable and needs earlier durability-focused escalation), while maintaining governance and interpretability suitable for real-world deployment. Second, it can move cardiometabolic drug development and implementation beyond trial-and-error by supporting biomarker-to-target translation and rational combination design in a network disease where single-node interventions are often insufficient and polypharmacy risk is real. The next critical step, and from our perspective the standard that should define success, is prospective, endpoint-specific validation showing that multiomic, mechanistic stratification improves calibration and decision-making, reduces incident T2D and cardiometabolic events, and does so equitably across diverse populations. In short, the value proposition is not “AI for prediction,” facilitating earlier detection, biologically stratification, and outcome-oriented intervention selection for the cardiometabolic continuum.

## Figures and Tables

**Table 1:** Multiomic Biomarker Classes in T2D-Centered Cardiometabolic Disease: Mechanistic Signals, Clinical Applications, and Translational Limitations.

Biomarker Class	Mechanistic Signal	Clinical Application	Key Limitations
**Genomics**	Inherited susceptibility; β-cell & insulin resistance pathways	Lifetime risk stratification; early prevention intensity	Ancestry bias; static signal; limited near-term calibration
**Epigenomics**	Metabolic memory; exposure imprinting; persistent regulatory bias	Long-horizon risk; complication susceptibility	Tissue specificity; causality; batch effects
**Transcriptomics**	Immune and stress-response activation; pathway state	Endotype identification; trajectory monitoring	Temporal variability; tissue specificity
**Proteomics**	Endocrine signaling; inflammation; endothelial injury; coagulation/complement	Residual ASCVD/HF risk refinement; monitoring	Platform harmonization; confounding (CKD/inflammation)
**Metabolomics**	Substrate flux; mitochondrial stress; amino-acid and lipid oxidation signatures	Preclinical progression prediction; response monitoring	Fasting/diet effects; cross-platform reproducibility
**Lipidomics**	Lipotoxicity; ceramide/sphingolipid signaling; remnant-rich phenotypes	Residual ASCVD risk; lipotoxic endotypes	Analytic complexity; standardization; causal attribution
**EV/Exosomes**	Inter-organ signaling; cargo remodeling under stress; pathway-linked modules	Early detection; monitoring; complication risk stratification	Isolation/characterization sensitivity; reporting standards required

## Data Availability

No new data were created or analyzed in this study. Data sharing is not applicable to this article.

## Supplementary Materials

The following supporting information can be downloaded at: https://www.mdpi.com/article/10.3390/ijms27093969/s1.

## Abbreviations

AI	artificial intelligence
ASCVD	atherosclerotic cardiovascular disease
CHD	coronary heart disease
CKD	chronic kidney disease
ER	endoplasmic reticulum
EV	extracellular vesicle
FPG	fasting plasma glucose
HbA1c	glycated hemoglobin
HF	heart failure
LDL	low-density lipoprotein cholesterol
MDASLD	metabolic dysfunction-associated steatotic liver disease
ML	machine learning
OGTT	oral glucose tolerance test
PK	pharmacokinetic
PD	pharmacodynamic
T2D	type 2 diabetes
