# Supplementary material for: Operon™ Platform-Enabled for Cardiometabolic Biomarker Screening and Precision Treatment Strategies: A Type 2 Diabetes-Centered Review with Cardiovascular Extension
Source: Int J Mol Sci. 2026 Apr 29;27(9):3969. doi: 10.3390/ijms27093969 (PMC13163407; doi:10.3390/ijms27093969)
Supplement: Supplementary file 1 [file ijms-27-03969-s001.zip › ijms-4190721-supplementary.pdf]

Supplementary Table S1. Representative multiomic studies in type 2 diabetes and cardiometabolic disease.

Study (Author, Year)	Cohort/Design	Omics Layers	Key Biomarkers/Findings	Clinical Application	Limitations
Li et al., 2025[1]	Prospective multi-cohort	Epigenomics, Proteomics, Metabolomics	Integrated signatures predicting T2D; pathway-linked targets	Early prediction; target identification	Complex integration; external validation needed
Bragg et al., 2022 (UK Biobank) [2]	Population cohort	Metabolomics (NMR)	Metabolic biomarkers improved T2D prediction	Risk stratification	Limited mechanistic attribution
Fraszczyk et al., 2022[3]	Meta-analysis (prospective cohorts)	Epigenomics	CpG sites associated with incident T2D	Early detection	Tissue specificity; causality unclear
Cheng et al., 2023[4]	Prospective cohorts	Epigenomics	Methylation risk scores improve prediction	10-year risk prediction	Population generalizability
GTEx Consortium, 2020[5]	Multi-tissue resource	Transcriptomics	Tissue-specific gene regulation	Mechanistic mapping	Limited disease-specific endpoints
Zeevi et al., 2015[6]	Prospective intervention	Microbiome + Clinical	Microbiome predicts glycemic response	Personalized nutrition	High variability; replication challenges
Collado et al., 2025[7]	Experimental + human validation	EV/Exosomes	EV arginase-1 → endothelial dysfunction	Mechanistic biomarker; CVD risk	Translational scalability
Carciero et al., 2024[8]	Review synthesis	EV/Exosomes	EVs mediate metabolic and inflammatory pathways	Biomarker framework	Standardization required
Jenkins et al., 2024[9]	AI validation study	AI modeling	AI predicts molecular activity from structure	Drug development support	Limited clinical endpoint validation
Lakey et al., 2025[10]	Narrative review + ML	Multiomics + AI	ML enables early T2D prognosis	Precision screening	Needs prospective validation

Supplementary Table S2. Summary of validation evidence and performance characteristics for the Operon™ mechanistic AI platform.

Study/Source	Design	Disease Context	Input Data Types	Output	Performance Metrics	Validation Type	Notes/Limitations
Study/Source	Design	Disease Context	Input Data Types	Output	Performance Metrics	Validation Type	Notes/Limitations
Internal validation (reported)	Blinded evaluation	Multi-disease (including T2D)	Multiomic + clinical	Biomarker classification	Sensitivity ~86%, Specificity ~91%	Retrospective	Controlled setting; endpoint-specific validation needed

AI molecular modeling	Preclinical/algorithm validation	Drug discovery	Chemical structure + biological data	Molecular activity prediction	High concordance reported	Internal/experimental	Limited clinical endpoint linkage
Platform integration studies	Retrospective analyses	Cardiometabolic	Multiomic datasets	Risk stratification, pathway mapping	Improved predictive performance vs baseline models	Retrospective	Heterogeneous datasets; requires prospective validation
Prospective deployment (conceptual)	Planned/ongoing	T2D, ASCVD, HF	Clinical + multiomic	Endotype classification, therapy guidance	TBD	Prospective	Critical for clinical translation

1. Li W, Cheng Y, Cui A, Huang M, Huang Q, Wang Q, Xia M, Qiu J, Peng Q, Li J, Li H, Wang Y, Zong G, Zheng Y, Wang J, Gao X, Ding C, Tang H, Jiang BH, Jin L, Li Y, Wang S. Multiomics Integration of Epigenetics, Proteomics, and Metabolomics Identifies Putative Drug Targets and Improves Early Prediction for Diabetes. Diabetes. 2025 Dec 1;74(12):2418-2431. doi: 10.2337/db25-0354. PMID: 40938633; PMCID: PMC12645165.
2. Bragg F, Trichia E, Aguilar-Ramirez D, Bešević J, Lewington S, Emberson J. Predictive value of circulating NMR metabolic biomarkers for type 2 diabetes risk in the UK Biobank study. BMC Med. 2022 May 3;20(1):159. doi: 10.1186/s12916-022-02354-9. PMID: 35501852; PMCID: PMC9063288.
3. Fraszczyk E, Spijkerman AMW, Zhang Y, Brandmaier S, Day FR, Zhou L, Wackers P, Dollé MET, Bloks VW, Gào X, Gieger C, Kooner J, Kriebel J, Picavet HSJ, Rathmann W, Schöttker B, Loh M, Verschuren WMM, van Vliet-Ostaptchouk JV, Wareham NJ, Chambers JC, Ong KK, Grallert H, Brenner H, Luijten M, Snieder H. Epigenome-wide association study of incident type 2 diabetes: a meta-analysis of five prospective European cohorts. Diabetologia. 2022 May;65(5):763-776. doi: 10.1007/s00125-022-05652-2. Epub 2022 Feb 15. PMID: 35169870; PMCID: PMC8960572.
4. Cheng Y, Gadd DA, Gieger C, Monterrubio-Gómez K, Zhang Y, Berta I, Stam MJ, Szlachetka N, Lobzaev E, Wrobel N, Murphy L, Campbell A, Nangle C, Walker RM, Fawns-Ritchie C, Peters A, Rathmann W, Porteous DJ, Evans KL, McIntosh AM, Cannings TI, Waldenberger M, Ganna A, McCartney DL, Vallejos CA, Marioni RE. Development and validation of DNA methylation scores in two European cohorts augment 10-year risk prediction of type 2 diabetes. Nat Aging. 2023 Apr;3(4):450-458. doi: 10.1038/s43587-023-00391-4. Epub 2023 Apr 6. PMID: 37117793.
5. GTEx Consortium. The GTEx Consortium atlas of genetic regulatory effects across human tissues. Science. 2020 Sep 11;369(6509):1318-1330. doi: 10.1126/science.aaz1776. PMID: 32913098; PMCID: PMC7737656.
6. Zeevi D, Korem T, Zmora N, Israeli D, Rothschild D, Weinberger A, Ben-Yacov O, Lador D, Avnit-Sagi T, Lotan-Pompan M, Suez J, Mahdi JA, Matot E, Malka G, Kosower N, Rein M, Zilberman-Schapira G, Dohnalová L, Pevsner-Fischer M, Bikovsky R, Halpern Z, Elinav E, Segal E. Personalized Nutrition by Prediction of Glycemic Responses. Cell. 2015 Nov 19;163(5):1079-1094. doi: 10.1016/j.cell.2015.11.001. PMID: 26590418.
7. Collado A, Humoud R, Kontidou E, Eldh M, Swaich J, Zhao A, Yang J, Jiao T, Domingo E, Carlestål E, Mahdi A, Tengbom J, Végvári Á, Deng Q, Alvarsson M, Gabrielsson S, Eriksson P, Zhou Z, Pernow J. Erythrocyte-derived extracellular vesicles induce endothelial dysfunction through arginase-1 and oxidative stress in type 2 diabetes. J Clin Invest. 2025 Mar 20;135(10):e180900. doi: 10.1172/JCI180900. PMID: 40111409; PMCID: PMC12077887.
8. Carciero L, Di Giuseppe G, Di Piazza E, Parand E, Soldovieri L, Ciccarelli G, Brunetti M, Gasbarrini A, Nista EC, Pani G, Pontecorvi A, Giaccari A, Mezza T. The interplay of extracellular vesicles in the pathogenesis of metabolic impairment and type 2 diabetes. Diabetes Res Clin Pract. 2024 Oct;216:111837. doi: 10.1016/j.diabres.2024.111837. Epub 2024 Aug 21. PMID: 39173679.
9. Jenkins I, Narayan I Vaishnavi, Uffens J, et al. AI-Based Predictions of Molecular Target Activity from Blind Chemical Structures. *Am J Biomed Sci Res*. 2024;23(1).

10. Lakey JRT, Casazza K, Lernhardt W, Mathur EJ, Jenkins I. Machine Learning and Augmented Intelligence Enables Prognosis of Type 2 Diabetes Prior to Clinical Manifestation. *Curr Diabetes Rev*. 2025;21(8):e010224226610. doi:10.2174/0115733998276990240117113408
